# Supplementary material for: Mechanically derived short-range order and its impact on the multi-principal-element alloys
Source: Nat Commun. 2022 Nov 9;13:6766. doi: 10.1038/s41467-022-34470-8 (PMC9646780; doi:10.1038/s41467-022-34470-8)
Supplement: Supplementary file 1 — Supplementary Information [file 41467_2022_34470_MOESM1_ESM.pdf]

SUPPLEMENTARY INFORMATION

Mechanically derived short-range order and its impact on the multiple-principal-element alloys

Jae Bok Seol[1,†,*], Won-Seok Ko[2,†], Seok Su Sohn[3], Min Young Na[4], Hye Jung Chang[4], Yoon-Uk Heo[5], Jung Gi Kim[1], Hyokyung Sung[6], Zhiming Li[7], Elena Pereloma[8] & Hyoung Seop Kim[5,*]

Correspondence and requests for materials should be addressed to J.B.S. (email: jb.seol@gnu.ac.kr) or to H.S.K. (email: hskim@postech.ac.kr)

Supplementary Figures

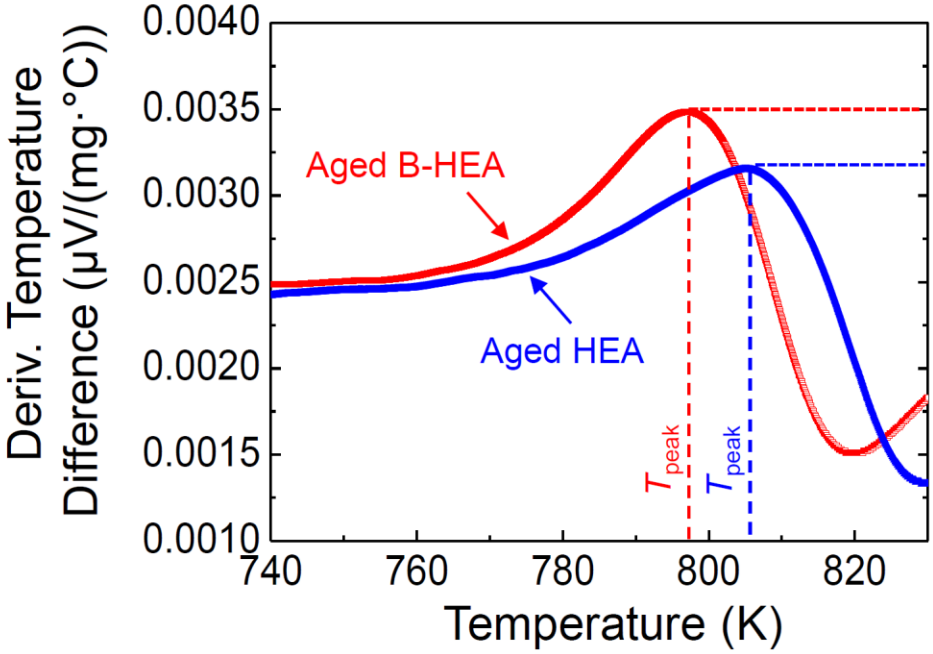

Supplementary Fig. 1 Thermal profiles obtained using the differential temperature analysis (DTA) technique. The red curve indicates the thermal history of the B-doped $Fe_{40}Mn_{40}Co_{10}Cr_{10}$ (at%) single-phase HEA sample (aged B-HEA, red curve) subjected to recrystallisation annealing and subsequent aging conditions that were followed by furnace cooling to room temperature. Also included is the DTA profile recorded for the interstitial-free version (HEA, blue curve) subjected to the same heat-treatment conditions. The heat-flow profiles recorded for the samples revealed the presence of exothermic peaks attributable to thermally activated CSROs. A detailed explanation of the profiles is described in Supplementary Notes 1. T_{peak}: peak temperature corresponding to the exothermic or heat-generation reaction.

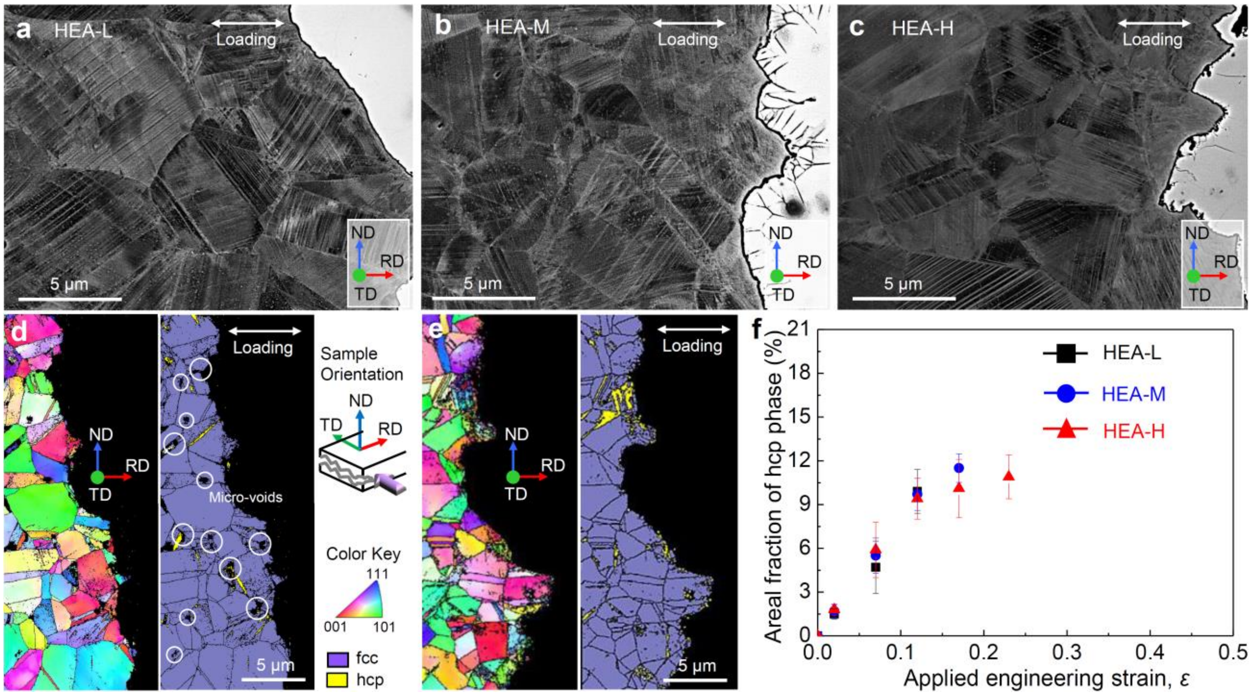

Supplementary Fig. 2 Microstructures of the tensile-tested samples tested at 77 K under conditions of varying strain rates. a, b Typical backscattered electron (BSE) images observed from the fracture surfaces of the HEA-L and HEA-M samples, respectively, where the tensile loading direction (arrows) is normal to the plane of view. **c** BSE image observed from the fracture surfaces of the HEA-H sample for comparison. **d, e** Corresponding EBSD images of the HEA-M (left panel) and HEA-H (right panel) samples. Micro-voids were formed along the grain boundaries, as marked by the green circles. In the EBSD-phase maps, violet and yellow scales represent fcc and hcp, respectively. **f** Areal fractions of the deformation-induced hcp martensitic phase as a function of applied strain, as determined from the EBSD-phase maps. The EBSD scanned area was ~30 × 300 μm^2. More than three EBSD scans were used to analyse the fractions.

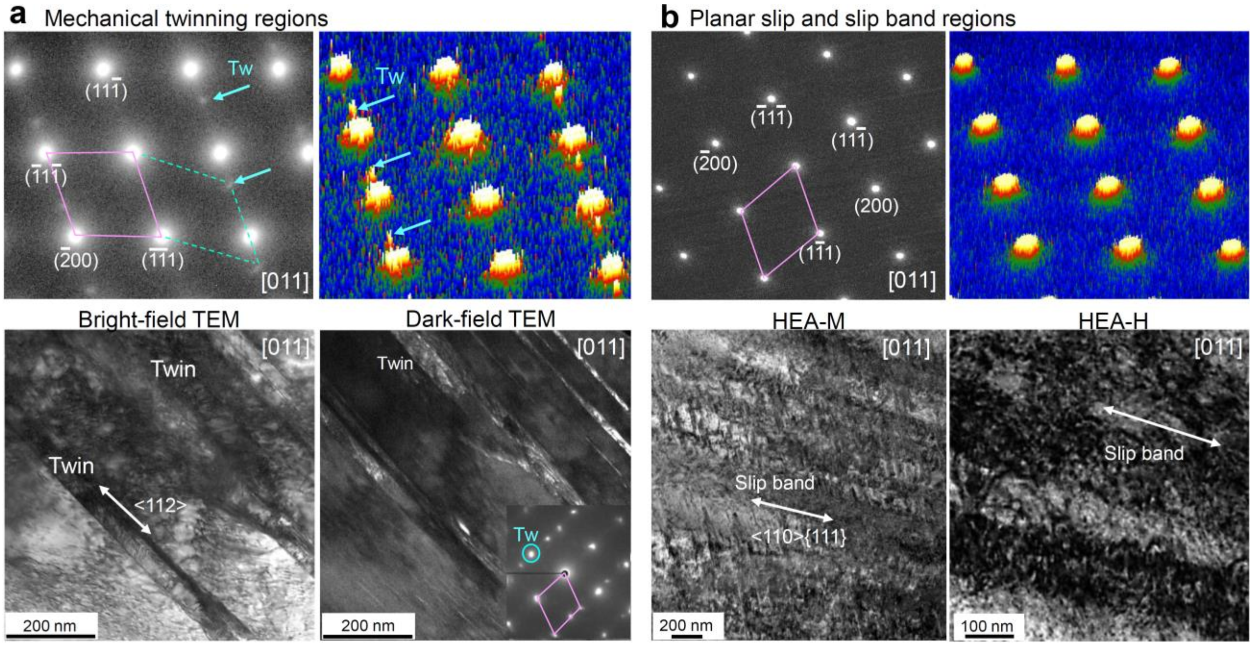

Supplementary Fig. 3 Comparison of TEM-EDPs from mechanical twinning and slip bands for the tensile-tested samples tested at 77 K. a Representative selected-area EDPs (top panel) and the corresponding coloured surface plots in three dimensions (mechanical twins; arrows and 'Tw' symbol). The plots present the colour scales with spot intensity on the electron diffractions (blue: background, green: low-intensity, yellow: high-intensity). Bottom panels: Representative TEM-bright field and dark-field images of mechanical twins in the deformation structure. **b** Representative TEM-EDP and corresponding for coloured surface plots. Bottom panels: Representative TEM images of slip bands (SBs) formed in the HEA-S (left panel) and HEA-M (right panel) samples. With an increase in the strain rates, the mean width of SBs that were inclined to the <110> directions lying on {111} planes decreased.

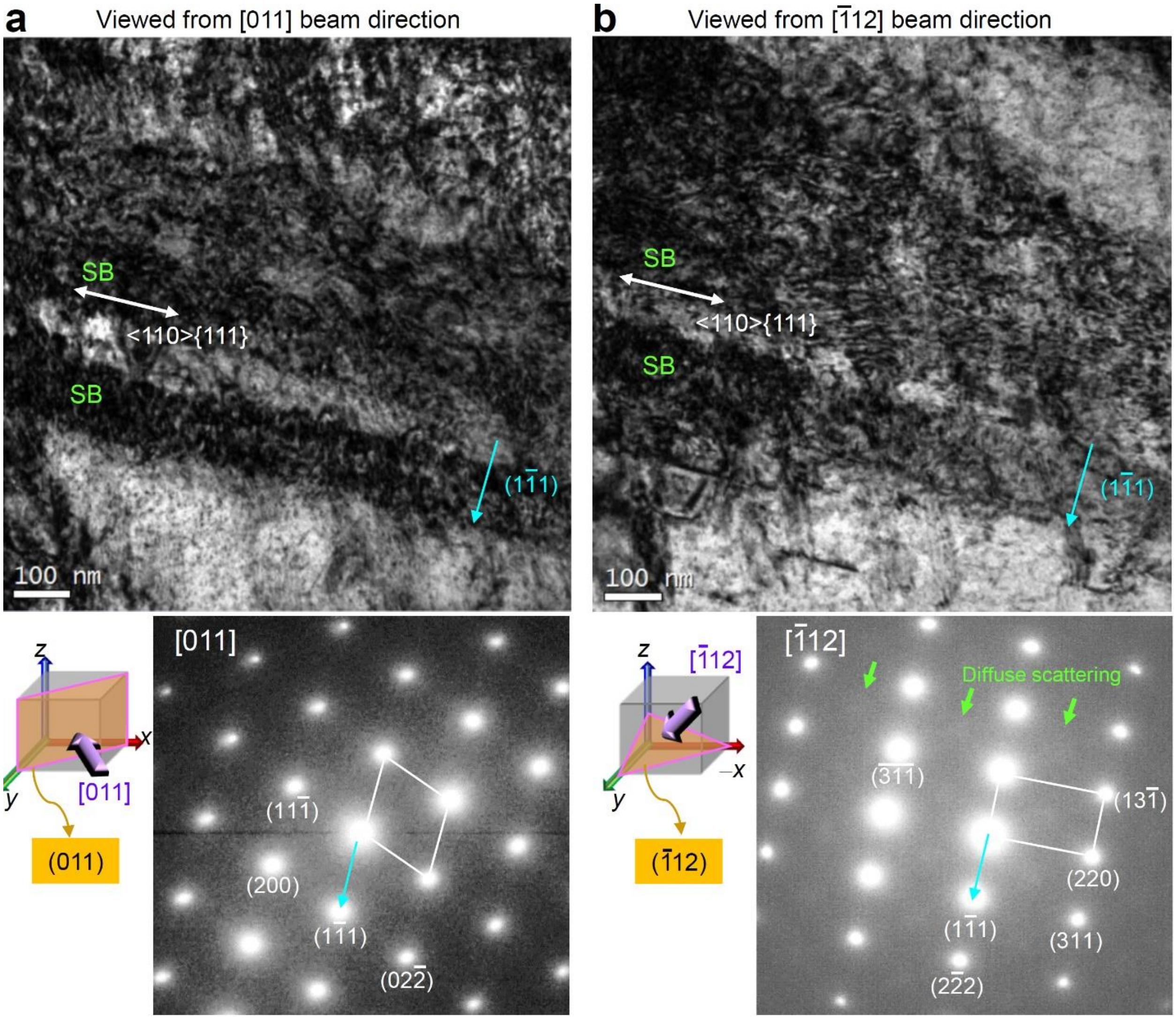

Supplementary Fig. 4 Substructure of SBs viewed from different electron beam directions. **a** TEM image (top) of the SBs and the corresponding EDPs (bottom) along the [011] zone axis (schematic illustration of the beam direction; left panel). **b** Another TEM-projected view along the [$\bar{1}$12] zone axis (schematic illustration of the beam direction; left panel). Under the [$\bar{1}$12] beam direction, TEM-EDP showed diffuse scattering at the ½{311} locations (green arrows). The images were recorded for the same TEM specimen but at different zone axes.

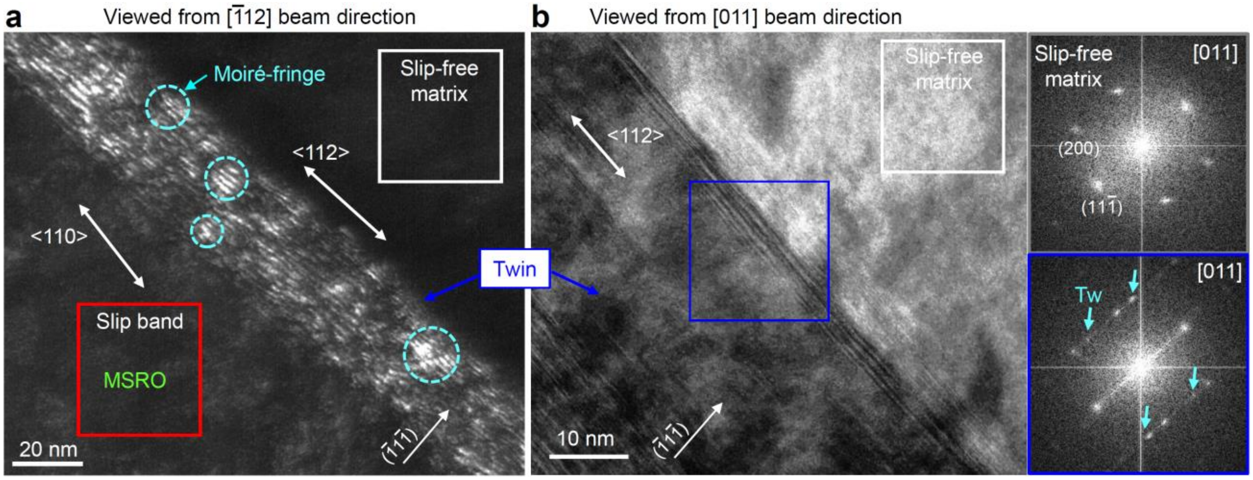

Supplementary Fig. 5 TEM image of Moiré fringes in twinned structures. a TEM dark-field image of strain-induced MSRO scattering along the [$\bar{1}$12] zone axis. In the SB-free regions, clustered order with bright contrast was not found (white box). Bright contrasts attributable to clustered MSRO domains (red box) were visible in the SB region. The TEM-artefact, the so-called Moiré-fringed lattice image attributable to double diffraction shown in Fig. 3e, was displayed at the overlapped area of SB and the twin, revealing the non-shearing event of MSROs. **b** TEM-projected view of the twinned structure along the [011] zone axis recorded using the HRTEM technique. The HRTEM image was recorded for the TEM specimen used in **a**. The corresponding FFT patterns, taken from the SB-free matrix (grey boxes) and twinned structure (blue boxes), revealing the presence of extra spots (attributable to mechanical twins (Tw)).

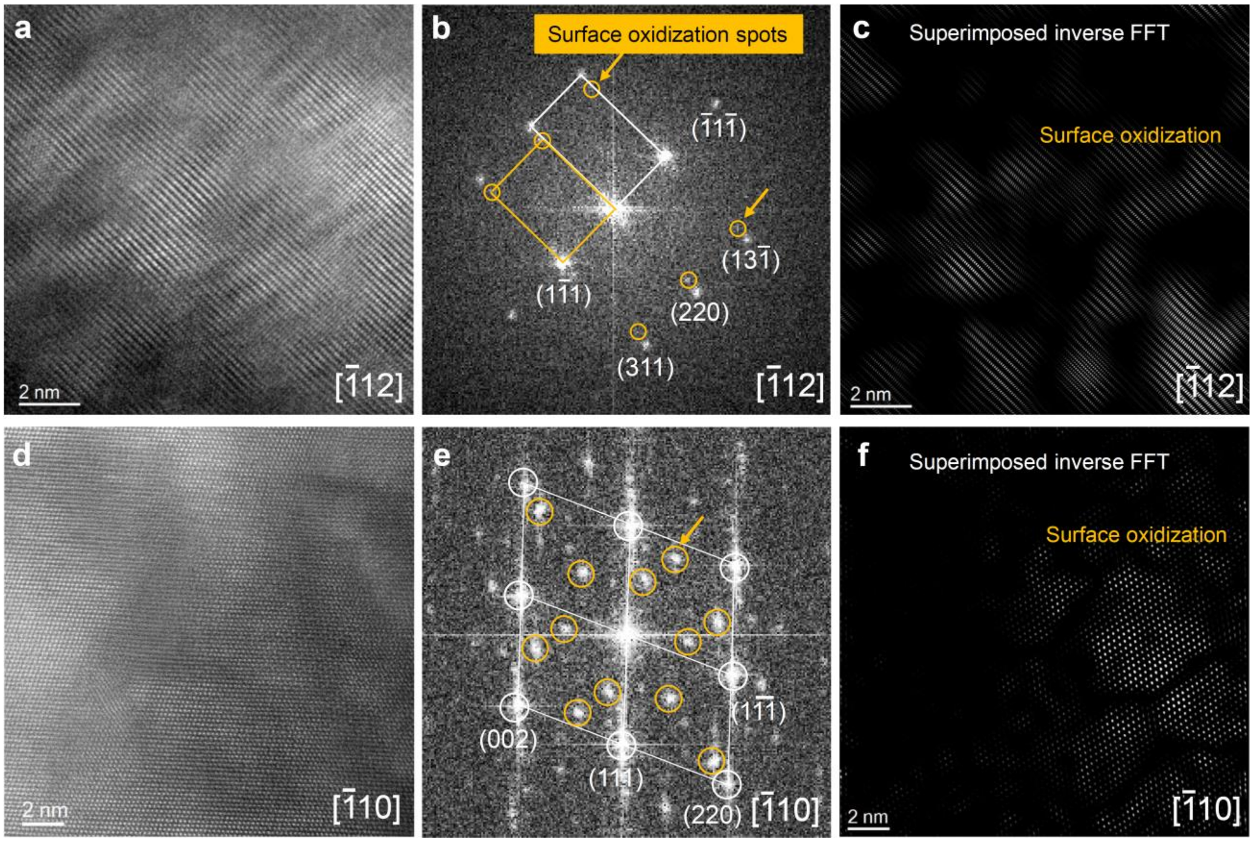

Supplementary Fig. 6 STEM images recorded for local surface oxidisation following in situ TEM heating tests. Top column: STEM images recorded along the [$\bar{1}$12] zone axis, bottom column: STEM images recorded along the [$\bar{1}$10] zone axis. The STEM images are obtained at the same sample position under different zone axes. **a, b** STEM image and corresponding FFT diffractogram of the in situ-heated TEM sample. The extra spots attributable to double diffraction, marked by the orange circles, near fundamental fcc Bragg spots indicate local surface oxidisation. **c,** Corresponding inverse FFT image obtained from oxide spots in **b**, revealing surface oxidisation. **d, e** STEM-HAADF image and the corresponding FFT diffractogram recorded for the in situ-heated sample, revealing local surface oxidisation (recorded for complex and layered oxide scales, including thermodynamically stable Cr_2O_3)[1] occurring during in-situ TEM tests. **f** Corresponding inverse FFT image obtained from oxide spots in **e**.

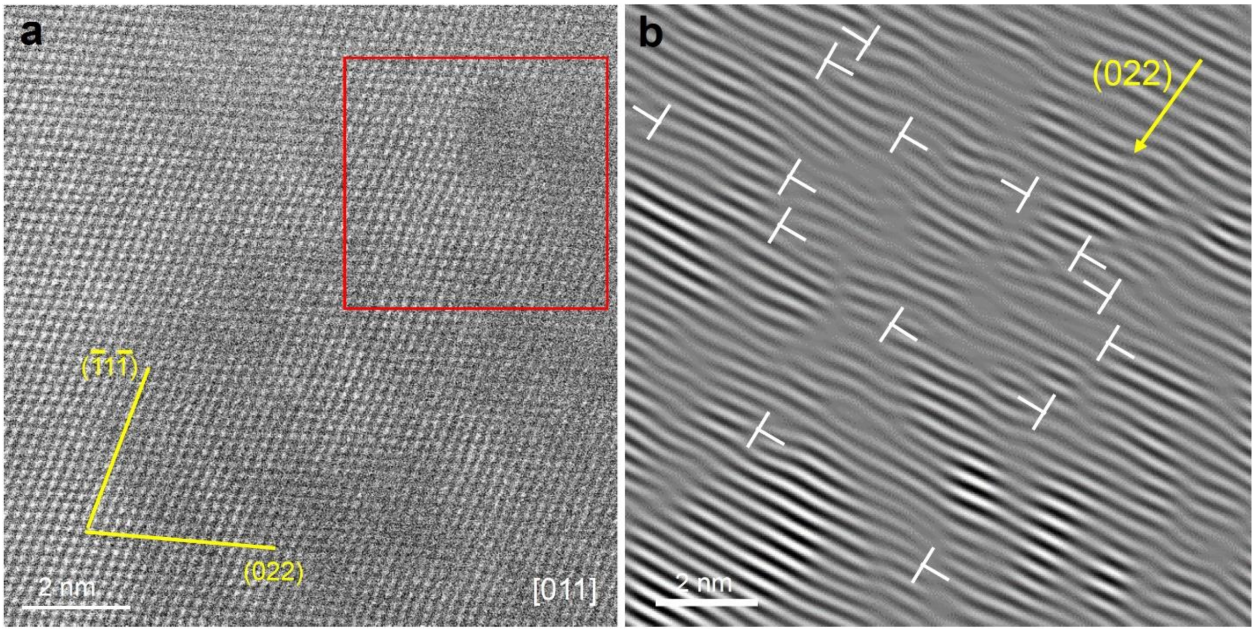

Supplementary Fig. 7 STEM image of lattice distortion recorded for the tensile-tested sample. a Representative STEM lattice image viewed from the [011] beam direction, taken from the HEA-M sample. **b** Corresponding inverse FFT (from the red box in **a**), revealing the abundance of full dislocations (‘T’ symbols).

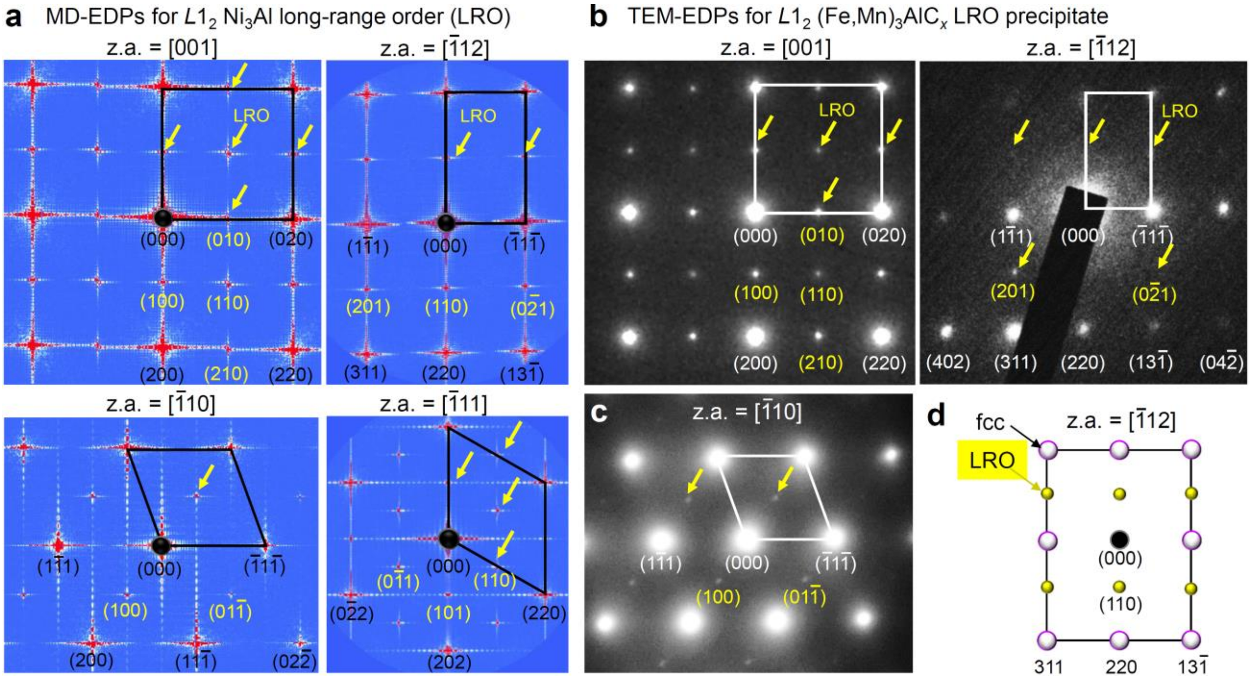

Supplementary Fig. 8 Comparison between the computationally obtained diffraction patterns and the experimentally obtained TEM diffraction patterns that were recorded from different zone axes for consistency. **a** MD-EDPs for $L1_2$ fcc-ordered Ni_3Al long-range order (LRO) structures along [001], $[\bar{1}12]$, $[\bar{1}10]$, and $[\bar{1}11]$ zone axes (z.a.). The LRO system presents extra diffraction spots, as indicated by the yellow arrows. **b, c** TEM-EDPs for $L1_2$-type fcc-ordered LRO precipitates in fcc-based high-Mn steel specimens with z.a. = [001], $[\bar{1}12]$[2], and $[\bar{1}10]$. **d** Schematic representation of the diffraction pattern indexed as $[\bar{1}12]$ z.a., where LRO generates superlattice reflections at the {110} and {201} locations in the TEM-EDPs. The MD-EDPs used in this work are consistent with the TEM-EDPs, outlining that the MD-EDPs can predict the experimentally obtained TEM-EDPs with several z.a.

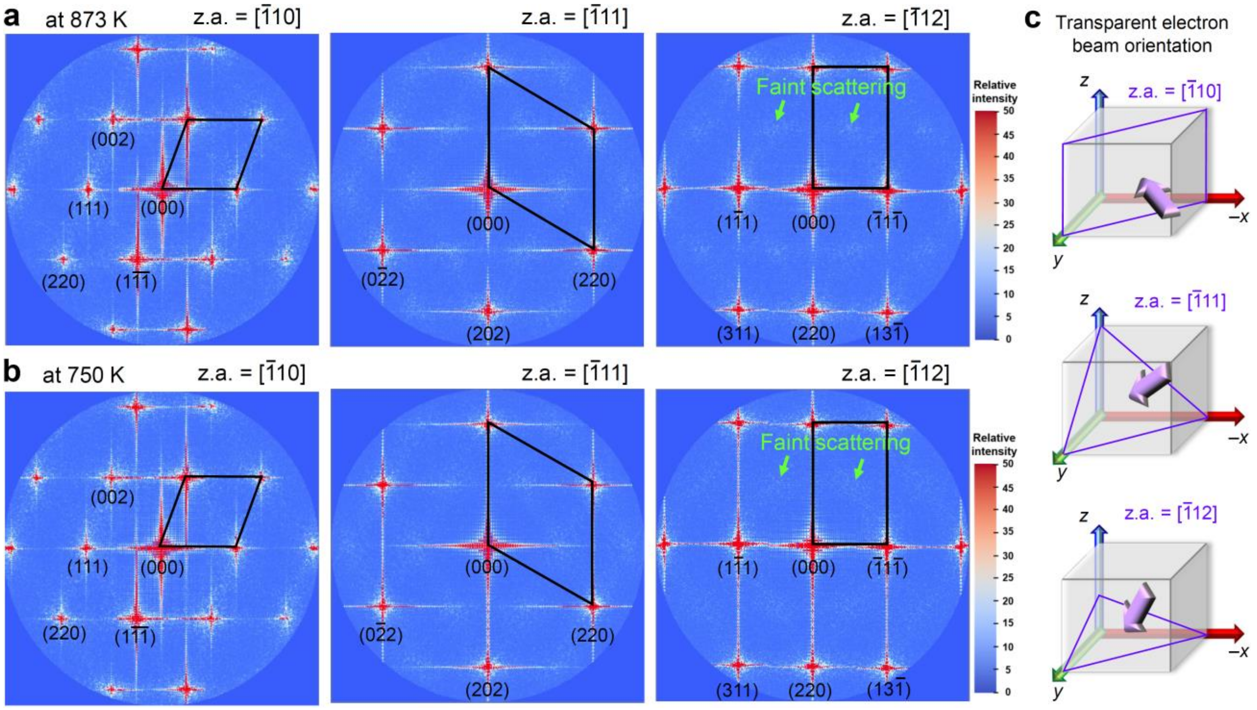

Supplementary Fig. 9 MD-EDPs for the $Fe_{40}Mn_{40}Co_{10}Cr_{10}$ (at%) solid solution, viewed along different zone axes. a MD-EDPs along the zone axes (z.a.) = [$\bar{1}$10], [$\bar{1}$11], and [$\bar{1}$12] for the interstitial-free HEA structure following atomistic MC simulation, showing the near-random distribution of the principal elements at 873 K (Fig. 1b). The colour scale (right corner) represents the relative intensity of the diffraction spots in arbitrary units (blue: low intensity; red: high intensity). **b** MD-EDPs for the simulated structure showing the irregular distribution of the principal elements following atomistic MC simulation at 750 K (Fig. 1c). We confirmed that the simulated EDPs corresponding to the HEA samples not subjected to conditions of atomistic MC simulations (in the absence of chemically ordered domains) were comparable to those obtained post MC simulation under CSRO-present conditions. **c** Schematic representation of the transparent electron beam orientations along the indicated arrows.

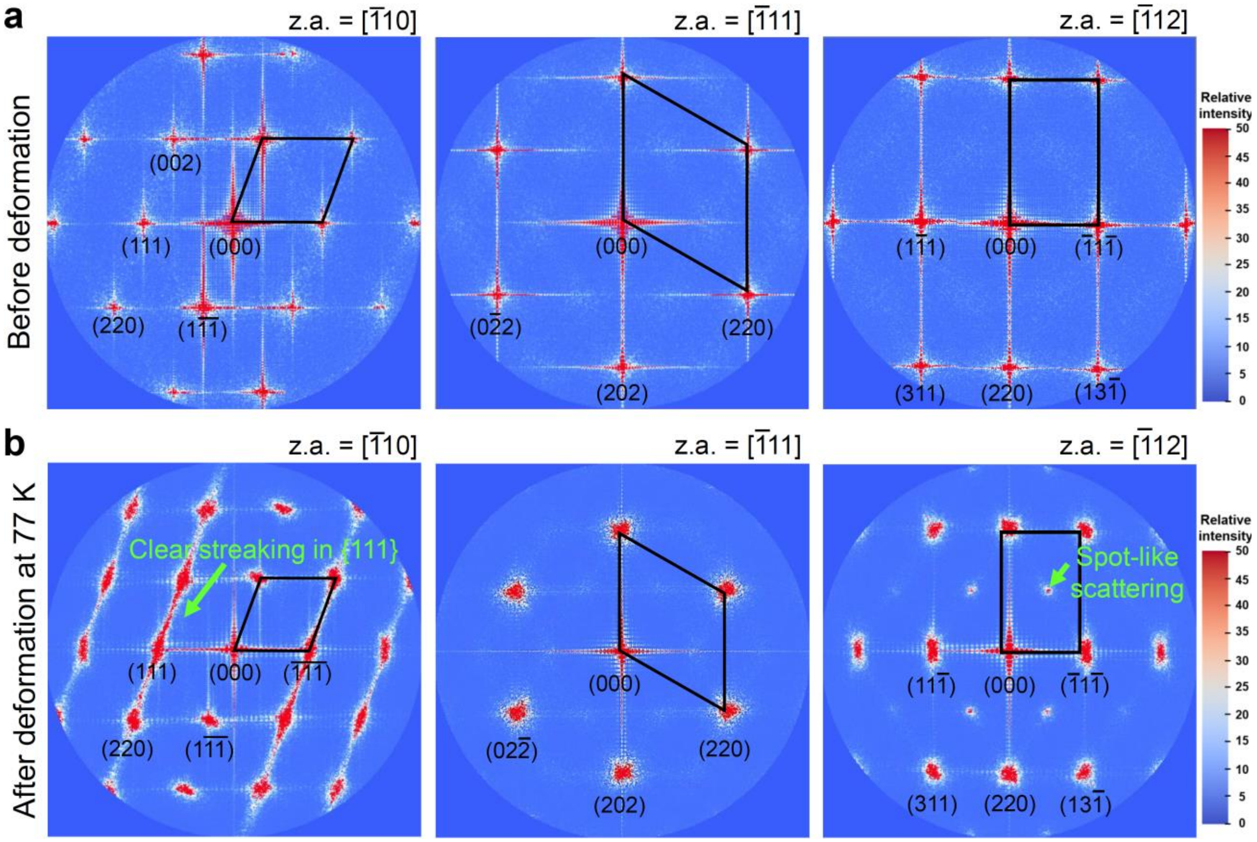

Supplementary Fig. 10 MD-EDPs for the $Fe_{40}Mn_{40}Co_{10}Cr_{10}$ (at%) HEA sample before and after deformation. a, b MD-EDPs for the HEA structure before and after deformation under different electron beam directions. The intensity of the strain-induced MSRO discs observed for the [$\bar{1}$12] EDP was higher than that observed for the [$\bar{1}$11] EDP. Clear streaking along the {111} directions for the fcc Bragg spots appeared in the [$\bar{1}$10] MD-EDPs, while the extra diffused discs in the [$\bar{1}$12] MD-EDPs appeared for a given structure. Streaking along the {111} directions for the [$\bar{1}$10] MD-EDPs and diffused discs corresponding to the [$\bar{1}$12] MD-EDPs were not observed.

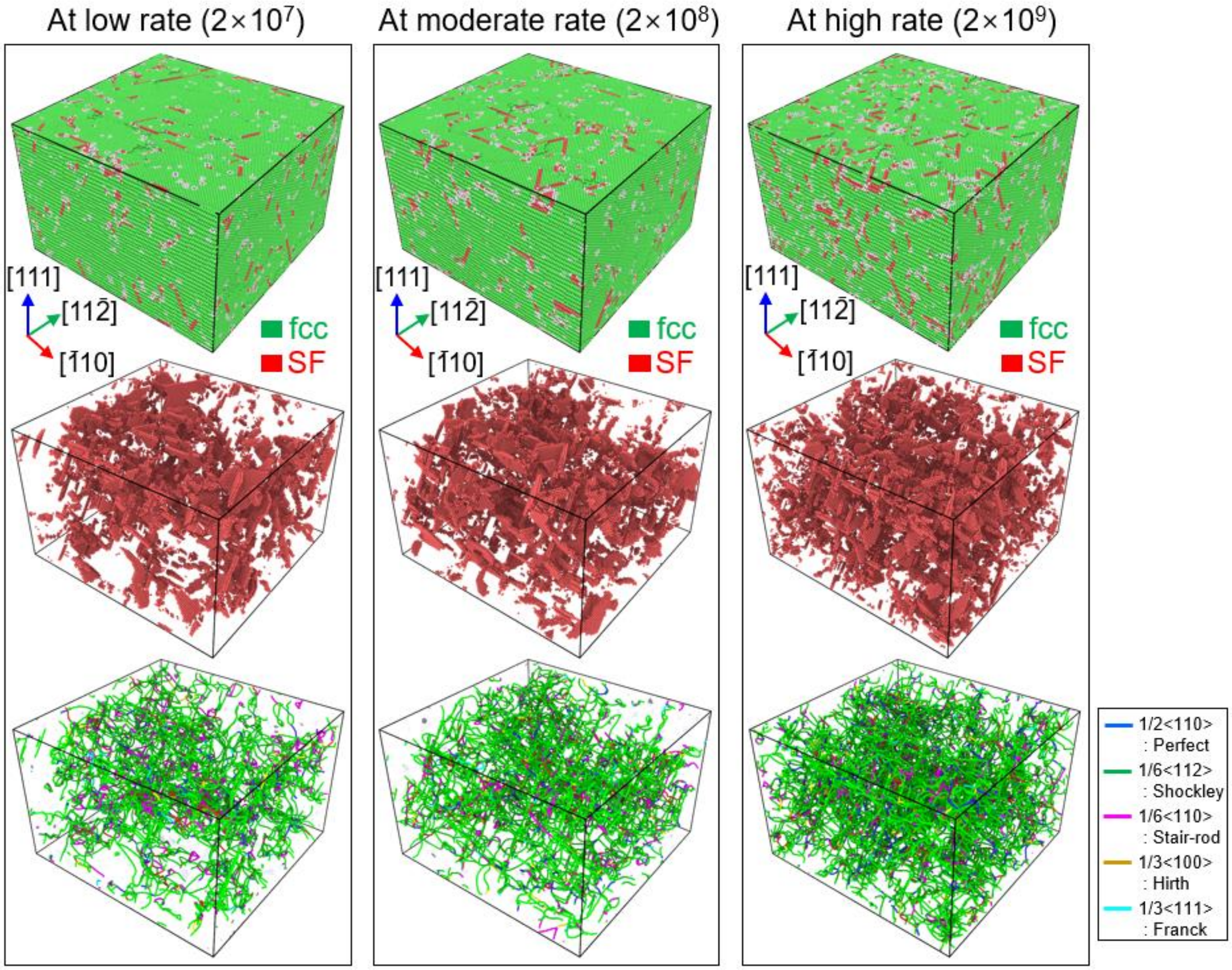

Supplementary Fig. 11 MD-simulated cell structures for the strained (at 77 K) $Ni_{60}Cr_{20}Co_{20}$ (at%) MEAs under conditions of varying loading rates. Changes in the density of dislocations with different Burgers vectors and in the SF fractions, with changes in the deformation rates for the $Ni_{60}Cr_{20}Co_{20}$ (at%) MEA with an SFE of 33.7 mJ/m^2. An increase in the rates results in an increase in the density of dislocations.

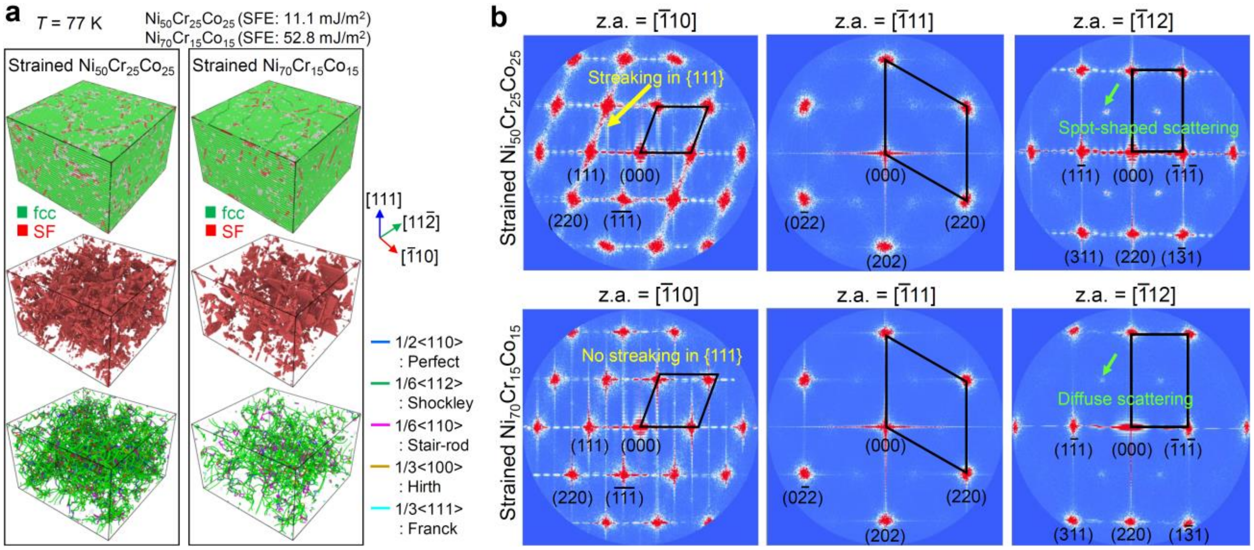

Supplementary Fig. 12 MD simulations for the strained MD-EDPs associated with Ni-based NiCrCo MEAs at 77 K. a Strained cell structures of the $Ni_{50}Cr_{25}Co_{25}$ and $Ni_{70}Cr_{15}Co_{15}$ (all in at%) samples with SFEs of 11.1 and 52.8 mJ/m^2, respectively, showing the distribution of deformation SFs and dislocations with different Burgers vectors. The deformation direction for each system was indicated along the [111]-direction. **b** MD-EDPs viewed along the [$\bar{1}$10], [$\bar{1}$11], and [$\bar{1}$12] electron beam directions for the deformed structures under conditions of medium strain rates. The MSRO-induced diffuse diffraction scattering (green arrows) increases with a decrease in the SFE. The spot-shaped MSRO scattering of high-intensity was seen in the [$\bar{1}$12] EDP for the low-SFE $Ni_{50}Cr_{25}Co_{25}$, while diffuse diffraction of low-intensity was observed in the EDP (z.a. = [$\bar{1}$12]) for the high-SFE $Ni_{70}Cr_{15}Co_{15}$ system. In addition, the intensity of the MSRO-induced discs in the [$\bar{1}$12] EDP system was higher than the intensity of the discs in the [$\bar{1}$11] EDP system. This trend was recognised for all the EDPs at a given alloy composition. Clear streaks among fcc Bragg spots were observed in the [$\bar{1}$10] MD-EDPs for the $Ni_{50}Cr_{25}Co_{25}$ system. These streaks were clearer than those observed for the $Ni_{70}Cr_{15}Co_{15}$ system.

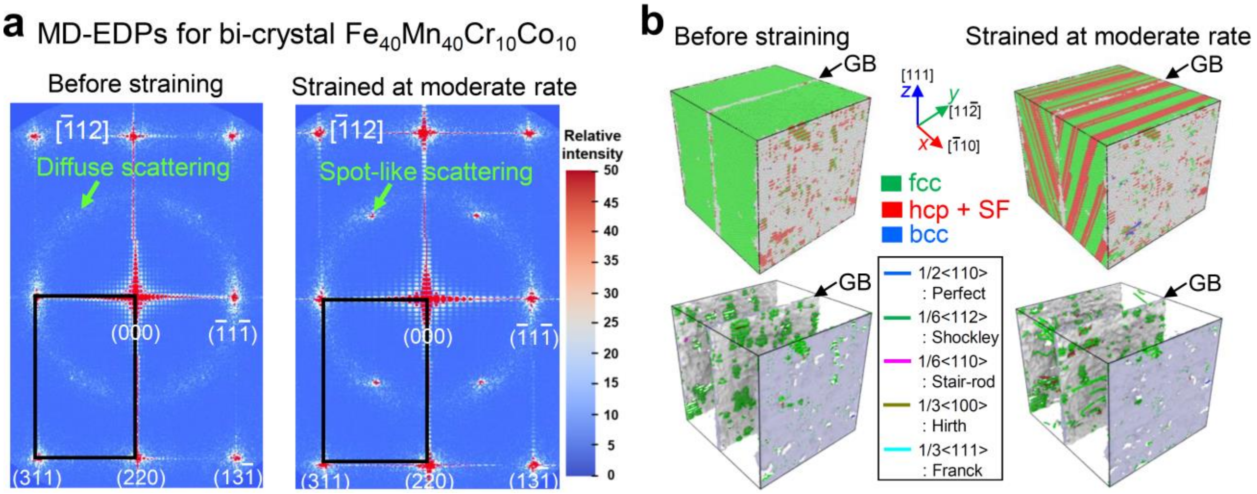

Supplementary Fig. 13 MD simulations for the origin of the MSRO-generated extra scattering in bi-crystal non-equiatomic FeMnCrCo HEA samples characterised by the presence of grain boundaries under conditions of loading at 77 K. **a** MD-EDPs for an interstitial-free $Fe_{40}Mn_{40}Cr_{10}Co_{10}$ (at%) structure before and after straining. The extra diffuse discs at the ½{311} locations in the [$\bar{1}$12] MD-EDPs become clearer when plastic strain is applied under conditions of moderate rate for a given structure. This is consistent with the result obtained for the single-crystal non-equiatomic FeMnCrCo HEA system (Fig. 6a). One-half of the bi-crystal cell was used for diffraction analysis following an MD run. **b** Corresponding bi-crystal cell structures, including a grain boundary (GB).

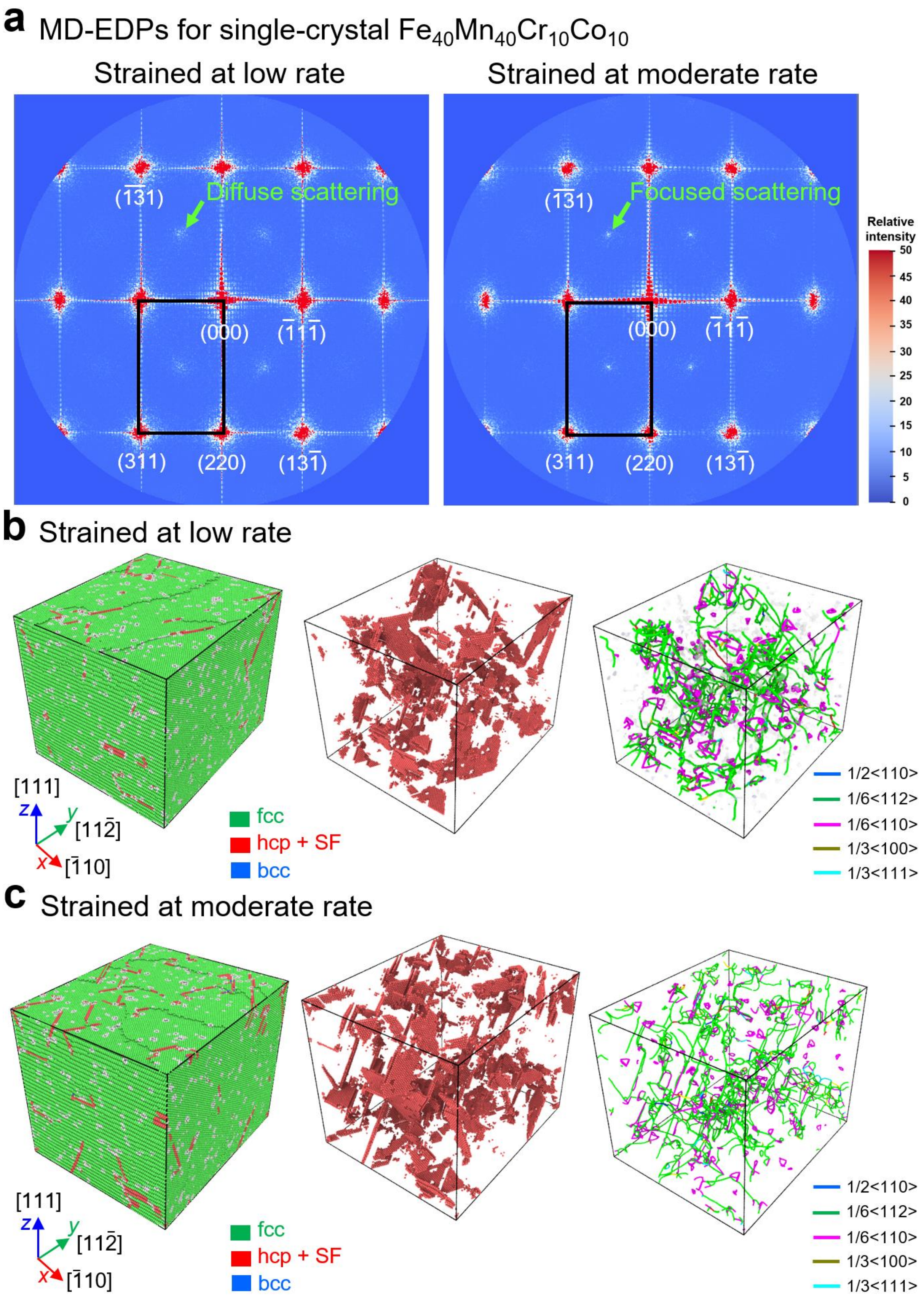
a MD-EDPs for single-crystal $Fe_{40}Mn_{40}Cr_{10}Co_{10}$
Strained at low rate
Strained at moderate rate
($\bar{1}\bar{3}1$)
Diffuse scattering
Focused scattering
(000)
($\bar{1}1\bar{1}$)
(311)
(220)
($13\bar{1}$)
Relative intensity
50
45
40
35
30
25
20
15
10
5
0
b Strained at low rate
c Strained at moderate rate
[111]
z
y
[$11\bar{2}$]
x
[$\bar{1}10$]
fcc
hcp + SF
bcc
1/2<110>
1/6<112>
1/6<110>
1/3<100>
1/3<111>

Supplementary Fig. 14 MD simulations, based on the LJ potential model, for single-crystal non-equiatomic FeMnCrCo HEA under conditions of loading at 77 K. a Evolution of MD-EDPs along the [$\bar{1}$12] zone axis for the HEA structure strained under conditions of low (left panel) and moderate (right panel) loading rates. Diffuse scattering at the ½{311} locations is shown, and the relative intensity of the diffuse scattering increases with an increase in the strain rate. These features obtained using the LJ potential model agree well with those obtained using other potential models (e.g., the 2NN MEAM) (Fig. 6a). **b**, **c** Corresponding cell structure strained under conditions of low and moderate loading rates, showing the distribution of deformation-induced SFs (middle panel) and dislocations with different Burgers vectors (right panel). An increase in the loading rate resulted in an increase in the slip planarity and SFs. The results agreed well with the results obtained using other potential models (e.g., the 2NN MEAM) (Fig. 6b).

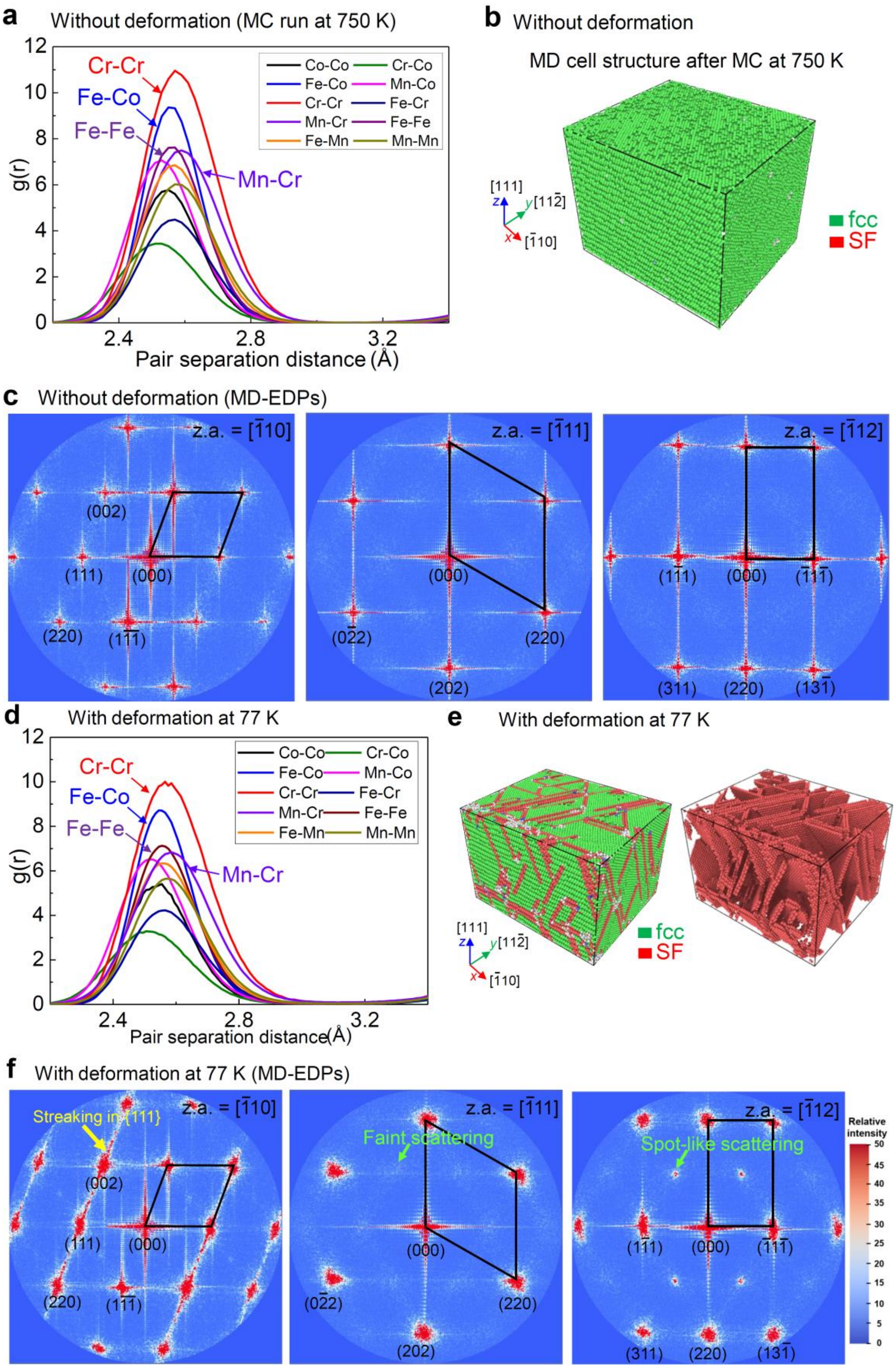
a Without deformation (MC run at 750 K)
b Without deformation
MD cell structure after MC at 750 K
fcc
SF
c Without deformation (MD-EDPs)
z.a. = [$\bar{1}$10]
z.a. = [$\bar{1}$11]
z.a. = [$\bar{1}$12]
d With deformation at 77 K
e With deformation at 77 K
f With deformation at 77 K (MD-EDPs)
Streaking in {111}
Faint scattering
Spot-like scattering
Relative intensity
Pair separation distance (Å)
g(r)

Supplementary Fig. 15 Atomic configurations, cell structures, and corresponding MD-EPDs observed along different zone axes for the fcc-structured $Fe_{40}Mn_{40}Co_{10}Cr_{10}$ (at%) HEA system before and after straining at 77 K. **a–c** Before straining (without MSRO). **d–f**, After straining at 77 K (with MSRO). Details are explained in Supplementary Discussion 4. **a, d** MC simulation-provided radial distribution function g(r) profiles of non-random distribution of the four principal elements in the HEA system without and with MSRO. The result presents Cr-enriched and Fe-Co-enriched features, revealing that the atomic configurations of MSRO are almost identical to those of CSRO. A cell with 216000 atoms (15.7 nm × 13.6 nm × 12.8 nm) prepared by conducting atomistic MC simulations at 750 K, shown in Fig. 1c, was used for straining at 77 K. The HEA structure, including the CSRO (Fig. 1c), was considered the initial state (without MSRO). **b, e** Corresponding MD simulation-based cell structures of the HEA system without and with the MSRO. The systems are visualised using the polyhedral template matching method (see Methods). **c, f** Corresponding MD-EDPs, viewed along different zone axes, of the HEA system without and with MSRO. Owing to the presence of MSRO in the MD cell structure, we observed not only streaking in the {111} slip planes under the $[\bar{1}10]$ zone axis but also clear extra spot-like ½{311} diffuse scattering under the $[\bar{1}12]$ zone axis in (**f**).

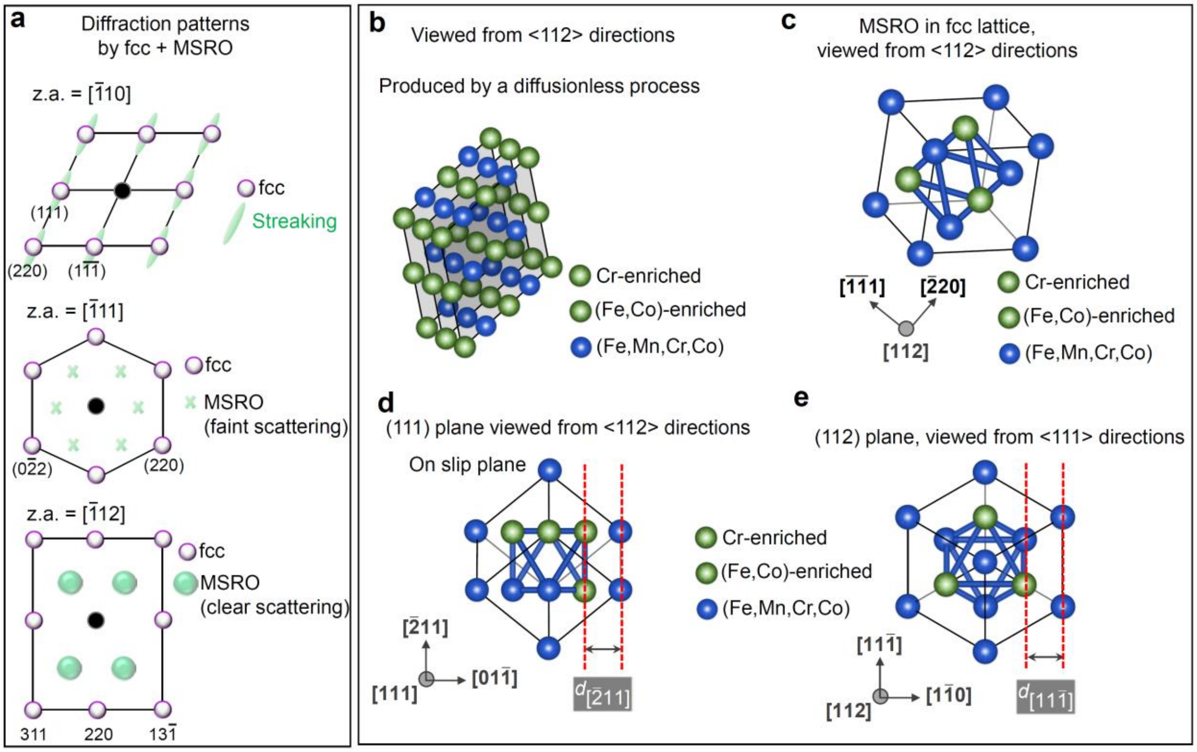

Supplementary Fig. 16 Schematic illustration of diffraction patterns and possible atomic configurations with MSRO in fcc-structured $Fe_{40}Mn_{40}Co_{10}Cr_{10}$ (at%) HEA system strained at 77 K. a Schematic representation of normal fcc reflections (grey circles) and MSRO scattering (presented in green). The TEM- and MD-EDPs were analysed under different zone axes. The EDPs described here agreed well with those associated with the fcc plus CSRO reported in literature[3-7]. **b** Motif of the atomic structure exhibiting MSRO (green circles) in the fcc structure (viewed along the <1 1 2> direction). The details are presented in Supplementary Discussion 4. **c** Possible 3D unit cells associated with MSRO along the <1 1 2> zone axis. **d, e** Corresponding 2D unit cells associated with MSRO on the $\{111\}_{fcc}$ slip plane under the $<112>_{fcc}$ directions, and that associated with MSRO on the $(112)_{fcc}$ plane under the $<111>_{fcc}$ directions. The results obtained using the TEM technique (Fig. 4e) revealed that the measured interplanar spacing (d_{MSRO}) corresponding to the MSRO {311} planes (green dotted lines) for the non-equiatomic HEA under study doubled the interplanar spacing (d_{fcc}) of the $\{311\}_{fcc}$ planes (white lines). This was similar to the case of CSRO in equiatomic MEAs[4,5]. A schematic representation of the unit cells has been presented.

Supplementary Table 1. **Stacking fault energies (SFEs) of fcc-based MPEAs.** SFEs were determined by conducting molecular statics simulation experiments at 0 K using 2NN MEAM and LJ interatomic potentials. The calculations were performed considering random fcc solid solutions at the compositions mentioned below.

System	Composition (at%)	SFEs (mJ/m^2)	Interatomic potential	Reference
FeMnCrCo HEA	$Fe_{40}Mn_{40}Cr_{10}Co_{10}$	-83.2	2NN MEAM	8
NiCoCr MEA	$Ni_{50}Co_{25}Cr_{25}$	11.1	2NN MEAM	8
	$Ni_{60}Co_{20}Cr_{20}$	33.7	2NN MEAM	8
	$Ni_{70}Co_{15}Cr_{15}$	52.8	2NN MEAM	8
FeMnCrCo HEA	$Fe_{40}Mn_{40}Cr_{10}Co_{10}$	25.3	LJ	9

Supplementary Notes

1. In principle, CSRO formation is a heat-generation process. Exothermic peaks in the DTA profiles indicate that the formation of CSRO in most solid solutions can be attributed to the strong negative ΔH_{mix} values (indicating heat generation). As shown in Supplementary Fig. 1, prominent exothermic peaks appeared in the profiles recorded for the heated samples. We found that the exothermic peak shifted to lower temperature regions, and this could be attributed to B doping (797 K, B-doped HEA; 806 K, B-free case). Moreover, the peak height increases with B ingress for a given heating rate (in the case of continuous heating). These results indicate the effect of a strong thermodynamic driving force for the formation of CSRO (attributable to B ingress). This validates the hypothesis that a large extent of Cr-rich CSROs can be introduced under B doping and ageing conditions.

2. We conducted tensile tests at 77 K under conditions of varying strain rates. We conducted the tests as the analysis of the TEM images recorded previously suggested that a decrease in the tensile testing temperature from 298 to 77 K resulted in the generation of deformation-induced order transition and resultant planar dislocation glides in boron-doped $Fe_{40}Mn_{40}Cr_{10}Co_{10}$ (at%) HEA[10]. However, the origin of the strain-driven SRO in the alloy could not be explained. The decrease in the tensile testing temperatures from 298 to 77 K can significantly affect the SFE values of the alloy, as SFE is a function of temperature. In other words, variations in the loading temperatures cannot indicate whether the generation of planar dislocation glides in fcc metallic alloys can be attributed to SRO or SFE. A clear consensus has not been reached on the origin of planar dislocation glide. This is primarily because SRO and SFE strongly influence the glide mode of dislocations and the hardening process associated with glide softening- or hardening-dominated fcc solid solutions. Chemical SRO is usually a thermally activated or diffusion-mediated process. This indicates that the formation of chemical SRO in MPEAs is sensitive to loading rates and temperatures, which can be attributed to the process of deformation-introduced heating. Hence, we selected the appropriate tensile testing parameters to minimise the effects of loading temperatures on the low SFE values. The parameters were also selected to minimise the extent of deformation-induced heating realised during tensile tests.

3. The MD-EDPs along the [001], [$\bar{1}$12], [$\bar{1}$10], and [$\bar{1}$11] zone axes for common $L1_2$-Ni_3Al structure are shown in Supplementary Fig. 8a. The predicted diffraction spots, attributed to the ordering in the structure, are highlighted by the arrows in all MD-EDPs. Especially, the MD-EDP along [$\bar{1}$12] MD-EDP is characterised by a zone axis that is suitable for directly imaging SRO (CSRO or MSRO) in the microstructure. The $L1_2$-structured LRO provides extra superlattice reflections at {201} (*i.e.*, halfway between the transmission spot (000) and the {402} spot) and {110} locations. The locations of the LRO-generated extra diffraction spots, determined by MD-EDPs, are consistently detected in the TEM-EDPs. This is even true for the [$\bar{1}$12] zone axis. The TEM-EDPs along the [001], [$\bar{1}$12][10], and [$\bar{1}$10] zone axes for a common $L1_2$ or $L'1_2$ LRO precipitate in high-Mn fcc-austenitic steel (Supplementary Fig. 8b). We suggest that the locations of MSRO-derived diffuse scattering in the TEM-EDPs are well predicted by the MD-EDPs.

Supplementary Discussion

1. The problems posed by the loading rates are related to the inherent limitation of atomistic simulations. The significantly high $\acute{\varepsilon}$ values associated with the MD simulation method can potentially result in high yields and flow stress values (associated with stress–strain response). This can result in an increase in the density of dislocations (by more than several orders of magnitude). The density of dislocations is higher than that obtained by analysing experimental results reported following a previous modelling method[11]. For example, the density of dislocations predicted by the MD simulations used by us was 10^{16}–10^{17} /m^2, while a density of ~10^{10} /m^2 was calculated based on the TEM experiments. Additionally, for the MD simulations, amorphisation was observed at the highest $\acute{\varepsilon}$ level for the $Fe_{40}Mn_{40}Cr_{10}Co_{10}$ HEA structure. This hindered the study of the effect of $\acute{\varepsilon}$ on the simulated cell structure under equivalent conditions. However, the use of a significantly small sample and narrow selected area (for the MD simulations) could negate the effect of the significantly high dislocation density. In this regard, it is an important point to compare the experimental and simulation results by ignoring the absolute size and rate of loading. It should be studied whether the simulation method can be used to reproduce the slip mechanism of the experiment. As shown in the figure, the MD simulation method used by us can well reproduce the slip mechanism via partial dislocations of the usual fcc metals, and the method depends on specific SFE values. The results reported in the manuscript provide insights into the qualitative agreement between experiments and simulations.

2. Most of the MD simulations were performed using an initial cell characterised by a single crystal fcc structure. This pristine condition disregards the situation where dislocations are nucleated through interactions with grain boundaries. We performed additional MD simulation experiments and virtual diffraction analysis for the deformation of the $Fe_{40}Mn_{40}Cr_{10}Co_{10}$ system using a bi-crystal model to ascertain the possibility of the generation of this effect. To analyse the diffraction properties along the $[\bar{1}12]$ zone axis, we selected a tilt grain boundary with a boundary plane of {112}, the rotation axis of <110>, and misorientation angle of 70.53° (i.e., {112}<110> incoherent twin boundary). One-half of the bi-crystal cell was used for the diffraction analysis following one MD run. The atomic configurations and resultant MD-EDPs are presented in Supplementary Fig. 13. The EDPs exhibited by the bi-crystal cell are similar to the

EDPs exhibited by the single-crystal cell (i.e., focused scattering is observed along the [$\bar{1}$12] zone axis). This demonstrates that the key results obtained by using the reported MD simulation method are independent of the presence or absence of GBs.

3. We performed additional simulation experiments based on the interatomic potential for the current HEA system (based on a pairwise LJ model) to understand the dependency of the MD results on the selection of interatomic potential[9]. This potential results in the generation of stacking fault energies that are higher than that generated by the 2NN MEAM potential[8] (Supplementary Table S1). We focused on the evolution of MD-EDPs along the [$\bar{1}$12] zone axis to understand the possible dependence of the deformation behaviour on the interatomic potential. The results support the primary conclusions presented by us. As shown in Supplementary Fig. 14, the results obtained by considering the simple pairwise LJ potential agree well with the results obtained by considering the 2NN MEAM potential (e.g., the advent of diffuse scattering, the loading rate dependence of the scattering intensity, and strong slip planarity upon a higher loading rate). Hence, we infer that the results obtained using the MD simulation methods (considering the 2NN MEAM potential) are independent of the choice of interatomic potential.

4. The high-resolution and scanning TEM images recorded at 77 K for the deformation structure are shown in Fig. 4c-f. The atomic configurations were considered the atomic configurations with and without MSRO while conducting the atomistic Monte Carlo (MC) and Molecular Dynamics (MD) simulation experiments, respectively. Unfortunately, the results obtained by conducting the MC simulation experiments revealed that the non-random distribution of the four principal elements (specifically, Cr-enriched and Fe-Co-enriched features) in the undeformed fcc HEA structure were similar to those associated with the deformed fcc HEA structure (Supplementary Fig. 15a and d, respectively). This can be attributed to a decreased extent of diffusion for solutes or a paucity of vacancies during mechanical loading at 77 K. The observations indicate that MSRO primarily originated from a diffusionless process. This primarily explains the differences in CSRO (i.e., CSRO originates from the diffusion-mediated redistribution of principal elements). Hence, we assume that despite mechanical deformation at 77 K, atomic configurations of MSRO are comparable to those of CSRO.

Analysis of the TEM images revealed the MSRO-induced ½{311} diffuse scattering in electron reciprocal-space EDPs under the [$\bar{1}$12] zone axis. (Fig. 3d, e, and Fig. 4d) and the MSRO atomic structure (Fig. 4e). The MSRO was formed by the redistribution of the principal elements, which originated from the mechanical stacking faults inside slip bands (Fig. 4g). This indicates that, like the CSRO in equiatomic MEAs[4,5], the measured interplanar spacing (d_{MSRO}) corresponding to the MSRO {311} planes (green dotted lines) in the non-equiatomic HEA doubles the interplanar spacing (d_{fcc}) associated with the $\{311\}_{fcc}$ planes (white lines). The doubled d_{fcc} accounts for the origin of extra diffuse scattering at the ½{311} locations in the electron reciprocal space. These results are supported by the results obtained by conducting MD simulation studies (after MC simulation at 750 K) and the corresponding MD-EDPs (Supplementary Fig. 15b, c, e, and f). Although the MC simulations used by us failed to indicate the differences in the real-space atomistic structures without and with MSRO, the results from MD simulations revealed clear differences in the cell structures before and after deformation at 77 K. The MD cell structure of fcc non-equiatomic HEA before deformation, *i.e.*, without MSRO, exbibit only normal fcc spots in the MD-EDPs (Supplementary Fig. 15b and c).

Post deformation, the MD cell structure with MSRO is characterised by abundant mechanical stacking faults (red; Supplementary Fig. 15e). In the corresponding MD-EDPs, we not only observed clear streaks along the fcc slip planes {111} under the [$\bar{1}$10] zone axis but also extra spot-like ½{311} diffuse scattering properties under the [$\bar{1}$12] zone axis. The simulation results agree well with the TEM-EDPs of the current FeMnCrCo HEA with MSRO. More interestingly, the TEM- and MD-EDPs described in this study agree well with the previously reported TEM results for the CSRO structure in VCoNi and CrCoNi MEAs. The similarity in the location of the ½{311} diffuse scattering associated with MSRO reveals that it is highly plausible that the real-space atomistic structure with MSRO in the FeMnCrCo HEA structure is identical to that of CSRO in VCoNi and CrCoNi MEAs. We note that the CSRO can be constructed by the $L1_1$-type structure motif in the fcc MEA structures[12]. The MSRO unit cell is likely to present an $L1_1$-type motif, and the atomic occupation in MSRO during mechanical loading at 77 K is re-arranged as a result of the diffusionless process, *i.e.*, stacking faults and edge dislocations inside the slip band. Hence, based on the concept of the CSRO motif, we provide a schematic representation of the MSRO atomistic structure motif (Supplementary Fig. 16). We sketched the diffraction patterns attributable to normal fcc spots and extra MSRO scattering based on the TEM- and MD-EDPs

along different zone axes (Supplementary Fig. 16a). The EDPs described herein agree well with those obtained for the fcc plus CSRO presented in the literature. Next, we constructed atomic projections of MSRO along different zone axes. The unit cells of the MSRO plus fcc structure were sketched based on the results obtained using the TEM technique (Fig. 4e), which revealed that the measured interplanar spacing (d_{MSRO}) associated with the MSRO {311} planes (green dotted lines) in non-equiatomic HEA doubled the interplanar spacing (d_{fcc}) of the $\{311\}_{fcc}$ planes (white lines). Similar observations were made for CSRO in equiatomic MEAs[4,5]. Supplementary Fig. 16b presents the schematic representation of the motif of the atomic structure exhibiting MSRO (green circles). The system was viewed from the <1 1 2> direction. Based on this motif, we present the simplified three-dimensional (3D) unit cell of MSRO in the fcc crystalline lattice along the <1 1 2> zone axis (Supplementary Fig. 16c). Furthermore, the possible 2D unit cell corresponding to MSRO on the $\{111\}_{fcc}$ slip planes under the $<112>_{fcc}$ direction (Supplementary Fig. 16d) and those corresponding to MSRO on the $(112)_{fcc}$ plane under the $<111>_{fcc}$ direction are schematically illustrated in Supplementary Fig. 16e.

Supplementary References

1. Schreiber, D. K. *et al*. Revealing the complexity of high temperature oxide formation in a 38Ni-21Cr-20Fe-13Ru-6Mo-2W (at. %) multi-principal element alloy. *Scr. Mater*. **210**, 114419 (2022).

2. Seol, J. B. *et al*. Short-range order strengthening in boron-doped high-entropy alloys for cryogenic applications. *Acta Mater*. **194**, 366–377 (2020).

3. Zhang, R. *et al*. Short-range order and its impact on the CrCoNi medium-entropy alloy. *Nature* **581**, 283–287 (2020).

4. Chen, X. F. *et al*. Direct observation of chemical short-range order in a medium-entropy alloy. *Nature* **592**, 712–716 (2021).

5. Zhou, L. *et al*. Atomic-scale evidence of chemical short-range order in CrCoNi medium-entropy alloy. *Acta Mater*. 224, 117490 (2022).

6. Wu, Y. *et al*. Short-range ordering and its effects on mechanical properties of high-entropy alloys. *J. Mater. Sci. Technol.* **62**, 214–220 (2021).

7. Liu, D. *et al*. Chemical short-range order in $Fe_{50}Mn_{30}Co_{10}Cr_{10}$ high-entropy alloy, *Mater. Today Nano.* **16**, 100139 (2021).

8. Choi, W. M., Jo, Y. H., Sohn, S. S., Lee, S. & Lee, B. J. Understanding the physical metallurgy of the CoCrFeMnNi high-entropy alloy: an atomistic simulation study. *Npj Comput. Mater*. **4**, 1–9 (2018).

9. Gröger, R., Vitek, V. & Dlouhý, A. Effective pair potential for random fcc CoCrFeMnNi alloys. *Model. Simul. Mater. Sci. Eng.*, **28**, 075006 (2020).

10. Seol, J. B. *et al*. Short-range order strengthening in boron-doped high-entropy alloys for cryogenic applications. *Acta Mater.* **194**, 366–377 (2020).

11. Ko, W. -S. *et al*. Atomistic deformation behavior of single and twin crystalline Cu nanopillars with preexisting dislocations, *Acta Mater.* **197**, 54–68 (2020).

12. Chen, X., Yuan, F. Zhou, H. & Wu, X. Structure motif of chemical short-range order in a medium-entropy alloy, *Mater. Res. Lett*. **10**, 149–155 (2022).
